# Light matters: testing the “Light Environment Hypothesis” under intra‐ and interspecific contexts

**DOI:** 10.1002/ece3.2188

**Published:** 2016-05-18

**Authors:** Angélica Hernández‐Palma

**Affiliations:** ^1^ School of Renewable Natural ResourcesLouisiana State University Agricultural CenterLouisiana State UniversityBaton RougeLouisiana

**Keywords:** Dichromatism, Furnariides, model of color discrimination, species recognition, tetrahedral color space model, visual signaling

## Abstract

The “Light Environment Hypothesis” (LEH) proposes that evolution of interspecific variation in plumage color is driven by variation in light environments across habitats. If ambient light has the potential to drive interspecific variation, a similar influence should be expected for intraspecific recognition, as color signals are an adaptive response to the change in ambient light levels in different habitats. Using spectrometry, avian‐appropriate models of vision, and phylogenetic comparative methods, I quantified dichromatism and tested the LEH in both intra‐ and interspecific contexts in 33 Amazonian species from the infraorder Furnariides living in environments with different light levels. Although these birds are sexually monochromatic to humans, 81.8% of the species had at least one dichromatic patch in their plumage, mostly from dorsal areas, which provides evidence for a role for dichromatism in sex recognition. Furthermore, birds from habitats with high levels of ambient light had higher dichromatism levels, as well as brighter, more saturated, and more diverse plumages, suggesting that visual communication is less constrained in these habitats. Overall, my results provide support for the LEH and suggest that ambient light plays a major role in the evolution of color signals in this group of birds in both intra‐ and interspecific contexts. Additionally, plumage variation across light environments for these drab birds highlights the importance of considering ambient light and avian‐appropriate models of vision when studying the evolution of color signals in birds.

## Introduction

Birds are highly visual animals; consequently, plumage color plays an important role in their communication and social signaling. Because of this, trying to understand the origins and maintenance of such color diversity has been an important theme in evolutionary biology and ecology (Hill and McGraw 2006b). Several hypotheses have been proposed to explain both intra‐ and interspecific variation of color in birds. For instance, sexual selection has been widely accepted as an explanation for the function and evolution of morphological differences between males and females (Cuthill et al. 1999), with sexually dichromatic species often assumed to have evolved from monochromatic ancestors by means of sexual selection for trait elaboration (Andersson 1994). Natural selection has also been proposed to explain sexual dichromatism, in which differences between sexes are linked to differences in predation risk, favoring cryptic females over “showy” ones (Owens and Hartley 1998). For interspecific variation, species recognition and risk of hybridization have been suggested as possible explanations; however, the “Light Environment Hypothesis” (LEH hereafter) has received more support (Marchetti 1993; McNaught and Owens 2002; Gomez and Thery 2004; Shultz and Burns 2013).

The LEH proposes that different species use different colors because they inhabit different light environments (Marchetti 1993). Thus, it can be used to predict what specific colors or levels of brightness would maximize (or minimize) contrast against the background, according to the amount of ambient light available in a particular habitat. Although originally proposed in the context of interspecific variation, this hypothesis can be used to explore intraspecific variation in relation to ambient light (Gomez and Thery 2004; Shultz and Burns 2013), because if ambient light has the potential to drive interspecific variation, a similar influence can be expected in relation to intraspecific recognition in birds living in different habitats.

As ambient light plays an important role in the evolution of color signals, it is important to account for variations in it and its potential effects on the evolution of plumage coloration, whether it is by constraining the conspicuousness of colors used for intraspecific signaling, or the efficiency of cryptic coloration meant to prevent detection by predators (Hill and McGraw 2006a; Gomez and Thery 2007). Forests exhibit highly dynamic light environments (Endler 1993), primarily due to the fact that vegetation at different strata strongly reduces light intensity (Hill and McGraw 2006a). Canopy and understory contrast drastically, with the former receiving more light, having higher spatial light diversity, and being richer in blue and ultraviolet (UV) wavelengths. In contrast, the understory receives considerably less light which is poor in UV but rich in greenish to yellow–green wavelengths (Endler 1993). Such contrasted environments offer a great opportunity for testing hypotheses of plumage color evolution in relation to ambient light. If visual signals are optimized, color differences should exist among and within species inhabiting different light environments.

Avian vision is highly specialized. Birds typically have four receptors that allow them to capture reflectance in the UV and near‐UV portion of the electromagnetic spectrum (Bennett and Cuthill 1994; Bowmaker et al. 1997; Hart et al. 1998; Cuthill et al. 2000). They also have specialized light‐filtering oil droplets that narrow the spectral sensitivity of each cone, enhancing their color discriminatory capabilities (Bowmaker 1980; Bowmaker et al. 1997; Hart et al. 1998). Furthermore, it has been well documented that birds, and vertebrates in general, possess mechanisms for color constancy, provided by means of the von Kries mechanism (von Kries 1905; Vorobyev et al. 2001; Stoddard and Prum 2008). Hence, despite environmental variation in ambient light spectral composition, bird visual capabilities should remain unaffected to a great extent (Stoddard and Prum 2008). However, recent studies investigating the influence of ambient light on the evolution of color signals in different groups have found significant associations between plumage coloration and habitat use (McNaught and Owens 2002; Gomez and Thery 2004, 2007; Shultz and Burns 2013).

In this study, I describe and analyze the influence of ambient light on the evolution of plumage color signals in 33 Amazonian bird species of the infraorder Furnariides (families Formicariidae, Dendrocolaptidae, and Furnariidae) living in environments with different levels of ambient light. Specifically, I want to test the LEH under both intra‐ and interspecific contexts. All the species in this analysis share a general plumage pattern with very similar colors and no sexual dichromatism perceptible to humans. Taking advantage of our current understanding of the visual system and color perception mechanisms in birds, I analyze color in a quantitative way which allows me to test the LEH in the context of color characteristics (including brightness).

If ambient light influences intraspecific variation, then I expect to see differences in the extent and amount of dichromatism found in species living under different light conditions. If this is the case, then communication needs to be enhanced at low light levels, which will be translated into more pronounced differences between males and females, whether in frequency or magnitude. The opposite would be expected in environments with higher light levels, as light is not expected to constrain visual signals in these habitats. Furthermore, if these differences have a role in sex recognition, then they should be mainly expressed in patches that are more readily visible to conspecifics.

With respect to interspecific variation, I expect birds signaling in the same light environment to have similar color characteristics. Support for the LEH will allow me to make predictions about the drivers of these differences. If differences are driven by sexual selection, then conspicuous signals that maximize contrast against the habitat background (vegetation) should be preferred. On the contrary, if natural selection is the driving force, color characteristics that enhance crypsis with the background should dominate the plumage of these birds. Given previous support for increased crypsis in other groups of birds, I predict that adaptation for crypsis should prevail. If this is the case, then measures of plumage color such as contrast, diversity, saturation, and brightness should be lower in birds living in low light environments.

The infraorder Furnariides is a large clade of about 600 species, endemic to the Neotropical region, that encompasses a diverse array of morphologies and behaviors (Moyle et al. 2009). The Furnariides have long been considered a cohesive evolutionary unit. All species in the group have a unique tracheophone syrinx, and monophyly of the group has been supported by molecular studies (Moyle et al. 2009). The clade shows an astonishing ecological diversity, occupying every terrestrial and water edge habitat in South America (Marantz et al. 2003; Remsen 2003). The ovenbirds (Furnariidae, 236 species), a true continental radiation (Claramunt 2010), show a tremendous diversity in ecomorphological adaptations including some extreme cases of morphological specialization among passerines (Remsen 2003). Woodcreepers (Dendrocolaptidae, 52 species) have advanced climbing adaptations to forage at all forest strata (Feduccia 1973; Remsen 2003). Ground antbirds (Formicariidae, 11 species), predominantly terrestrial forest birds with short wings and tails, are well adapted to feed on invertebrate prey near or on the ground (Krabbe and Schulenberg 2003). As mentioned earlier, a general plumage pattern is shared within the group. Almost all species are dull and sexually monochromatic to humans, with light brown to reddish‐brown body plumage and various degrees of spotting or streaking on the breast and back, often with light throat patches (Feduccia 1973; Krabbe and Schulenberg 2003; Remsen 2003). The great diversity of habitats occupied by the Furnariides, coupled with the plumage pattern shared by the group, the predominance of sexually monochromatic species, and its well‐known phylogeny, makes this clade ideal for testing hypotheses about the evolution of visual signals in relation to ambient light.

## Materials and Methods

### Species selection

**Figure 1:**
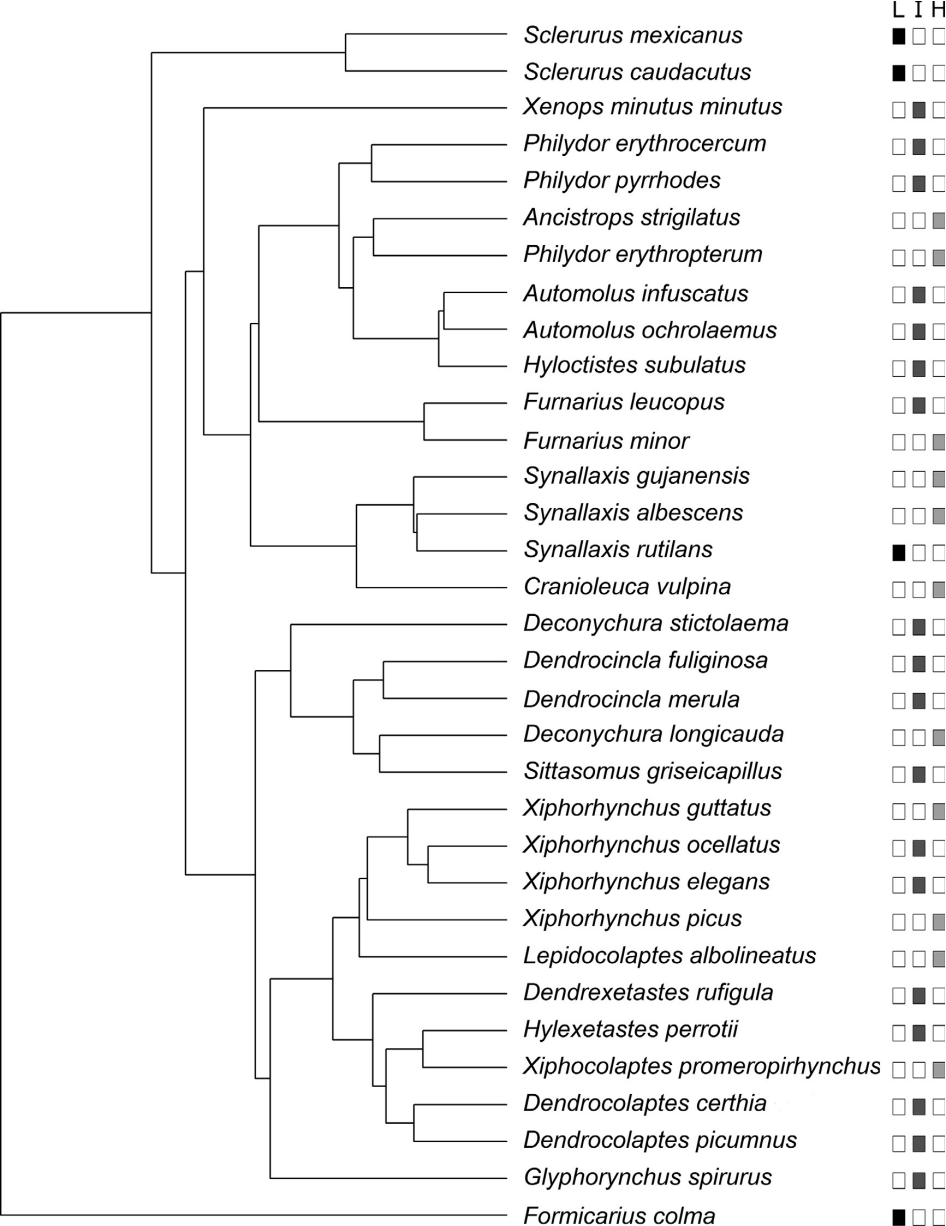
Phylogeny of 33 monochromatic species from the infraorder Furnariides (adapted from Derryberry et al. 2011). Black, dark gray, and light gray refer to the amount of ambient light available in each species' habitat: low (L), intermediate (I), high (H), respectively, following Stotz et al. (1996).

I selected 33 sexually monochromatic species (based on human standards of avian coloration) from the infraorder Furnariides (Fig. 1), living in Amazonian environments with different light levels, from the floor of dense *terra firme* forest to completely open habitats. This group is widely represented in Amazonian habitats as well as in the collection at the Louisiana State University Museum of Natural Science (LSUMNS); therefore, I selected species from across the phylogeny based on availability of specimens at the LSUMNS. In order to evaluate the influence of ambient light on the evolution of plumage color signals, I classified species into three groups using information on preferred habitat and foraging strata from Stotz et al. (1996). Species from the floor of *terra firme* forest were included in the low light group. The intermediate light group consisted of species from the understory and mid‐story of both *terra firme* and *várzea* forests. Finally, the high light group included species from the mid‐story and canopy of *terra firme* forest, and from all strata of river‐edge and second growth forests.

### Plumage color measurement

I collected reflectance spectra using an Ocean Optics USB2000+ spectrometer with a PX‐2 pulsed xenon lamp. For each species, I measured between 3 and 5 adult (ossified skull, no bursa) specimens of each sex, from the same locality and subspecies, when possible. I measured reflectance spectra from 5 to 10 plumage patches (>4 mm^2^), following designations widely used in the current ornithological literature: throat, breast, belly, crown, nape, back, rump, tail, wing coverts, and facial marks when present. Finely barred, streaked, or mottled plumage patches were ignored consistent with recommendations from Eaton (2005). Reflectance spectra were measured three times per patch; therefore, each reading is an average of three measurements per patch between 300 and 700 nm.

### Spectral data analyses

**Figure 2:**
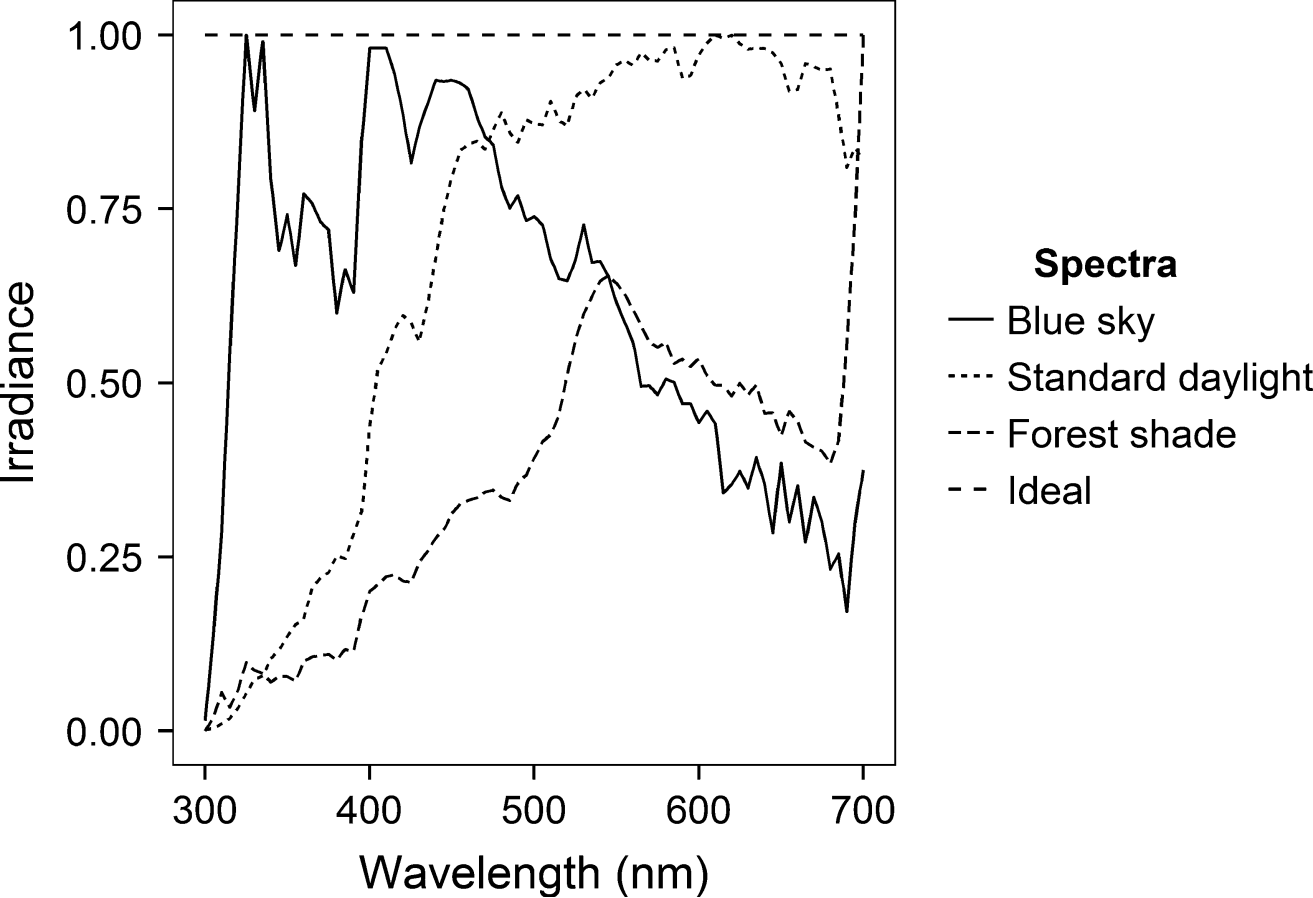
Irradiance spectra used for the Vorobyev–Osorio model of color discrimination (see “Materials and Methods” for details). Total irradiance (in the wavelength range 300–700 nm) for these illuminants is forest shade: 142.71, standard daylight: 275.87, blue sky: 249.10 *μ*mol·m^−2^·s^−1^ (data from Endler 1993).

To evaluate potential differences between males and females of the same species, I used the Vorobyev–Osorio (1998) model of color discrimination. This model calculates a distance in avian color space (Δ*S*, expressed in jnd –just noticeable differences) between homologous male and female patches, using the quantum catches of each cone cell type in the avian retina and their corresponding noise‐to‐signal ratios. To assess the effect of ambient light in color discrimination between sexes, I ran the model twice using different parameters. In both cases, I used the spectral sensitivity of the average avian UV system (Endler and Mielke 2005), an idealized homogenous background (white), a Weber fraction (*w*_i_) of 0.05 (for the most abundant cone type), and the cone densities of the blue tit (*Cyanistes caeruleus*). For the first set of models, I used ideal homogenous illuminance of 1 across all wavelengths (ideal model). For the second set of models, I used irradiance spectra appropriate to the ambient light experienced by each group, in order to explore the effects of varying levels of ambient encountered by species in their respective habitats (real model). I used forest shade irradiance for the low light group, standard daylight (D65) irradiance for the intermediate light group, and blue sky irradiance for the high light group. Even though the standard daylight irradiance has a higher total light intensity than the blue sky, I decided to use the latter one for the high light group because its intensity peaks in the UV region, similar to the forest canopy light environment (Fig. 2) (Endler 1993). Additionally, in order to understand the effect of ambient light spectra in the results obtained from the Vorobyev–Osorio model of color discrimination, I rerun the models for each species using all of the different irradiance spectra used for the other groups. Following Eaton (2005), I defined a feather patch as dichromatic when Δ*S* ≥ 1.0 and a species as dichromatic if it had at least one dichromatic feather patch. I also considered dichromatism under Δ*S* ≥ 1.5 and 2.0 to investigate changes in dichromatism levels with more conservative thresholds. Lastly, I performed an analysis of variance with group as blocks, to explore potential differences in discrimination values (Δ*S*) among groups and feather patches.

For the interspecific analysis, I used the tetrahedral color space model (Stoddard and Prum 2008), which provides a convenient, quantitative representation of bird color in a straightforward way relevant to bird vision with few assumptions (Stoddard and Stevens 2011). As with the model of color discrimination, I used the spectral sensitivity of the average avian UV system to calculate relative quantum catches, and ideal homogenous background and illuminance. I calculated six color characteristics for each species (males and females separate): average color span, the average of the Euclidean distances between each pair of colors in the plumage, and its variance; volume of color space, the volume of the minimum convex polygon that contains all the color points in the plumage; average and maximum chroma, and average brightness. Average brightness was calculated from the raw spectral data as the mean reflectance over the entire spectral range, following Montgomerie (2006). I performed an analysis of variance with sex as blocks, to explore potential differences in the color characteristics between males and females.

### Comparative phylogenetic analyses

I examined the evolution of color signals using the phylogeny of the clade by Derryberry et al. (2011), which was based on sequencing of three mitochondrial genes and one nuclear intron (Derryberry et al. 2011). To test whether plumage evolution is influenced by ambient light, I compared three models of evolution for each color descriptor: a Brownian motion model (BM), an Ornstein–Uhlenbeck model (OU) with a single optimum, and an OU model with three selective regimes based on the amount of ambient light available at each habitat (low, intermediate, high). I then compared the models using the AIC criterion corrected for small sample size (AIC_c_). When ΔAIC_c_ < 4, I used a likelihood ratio test to test for significant differences with simpler models; however, this could not be carried out when the less‐complex model had the lower AIC_c_. In cases when ΔAIC_c_ < 2, I considered the simpler model as the best model, because the additional parameters do not explain enough variation to be included in the model (Arnold 2010). Finally, I estimated the respective parameters for the best‐fit model of each descriptor from 10,000 bootstrap replicates.

I performed all analyses in R (R Development Core Team 2015) using the Pavo (Maia et al. 2013), Ape (Paradis et al. 2004), Geiger (Harmon et al. 2008), and Ouch (King and Butler 2009) libraries.

## Results

### Intraspecific variation

Under both the real and ideal models of color discrimination, 27 of 33 species (81.8%) were dichromatic in at least one plumage patch when Δ*S* ≥ 1.0 (Table 1). However, when the threshold for discrimination was doubled (Δ*S* ≥ 2.0), the number of dichromatic species dropped to 9 and 10 (27.3%, 30.3%) under the real and ideal models, respectively (Table 1). Six species *Glyphorynchus spirurus*,* Dendrexetastes rufigula*,* Dendrocolaptes certhia*,* Automolus infuscatus*,* Xiphorhynchus picus*, and *X. guttatus* were completely monochromatic at all thresholds of discrimination. The proportion of dichromatic species varied with levels of ambient light (Table 1). Under the real model, 100% of the species from the low light group were dichromatic at Δ*S* ≥ 1.0, but only 75% and 25% at the 1.5 and 2.0 thresholds. In the intermediate light group, 77.8% of the species were dichromatic at Δ*S* ≥ 1.0, but only 22.2% and 11.1% at the 1.5 and 2.0 thresholds. A less dramatic trend was observed in the high light group, with 81.8% of the species having at least one dichromatic patch at the Δ*S* ≥ 1.0 and 1.5 thresholds, and 54.5% at the Δ*S* ≥ 2.0 threshold.

**Table 1:** Number of dichromatic patches obtained under the ideal and real Vorobyev–Osorio model of color discrimination for 33 bird species of the infraorder Furnariides living in Amazonian habitats with different levels of ambient light. Three different discrimination thresholds were evaluated for each model (Δ*S* ≥ 1.0, 1.5, 2.0). Species in bold were identified as completely monochromatic at all levels of discrimination

Light level	Species	No. patches measured	Number of dichromatic patches					
			Real model			Ideal model		
			1.0	1.5	2.0	1.0	1.5	2.0
Low	*Formicarius analis*	10	6	4	0	6	4	1
	*Sclerurus mexicanus*	9	6	2	1	7	4	1
	*Sclerurus caudacutus*	9	1	1	0	1	1	0
	*Synallaxis rutilans*	9	1	0	0	1	0	0
Intermediate	*Dendrocincla merula*	9	1	0	0	1	0	0
	*Dendrocincla fuliginosa*	9	1	0	0	3	0	0
	*Deconychura stictolaema*	6	2	0	0	4	1	0
	*Deconychura longicauda*	6	1	0	0	1	0	0
	*Sittasomus griseicapillus*	9	2	0	0	2	0	0
	***Glyphorynchus spirurus***	8	0	0	0	0	0	0
	*Xiphorhynchus ocellatus*	6	1	1	0	3	2	0
	*Xiphorhynchus elegans*	6	1	0	0	2	0	0
	***Dendrexetastes rufigula***	9	0	0	0	0	0	0
	***Dendrocolaptes certhia***	5	0	0	0	0	0	0
	*Dendrocolaptes picumnus*	9	1	0	0	1	0	0
	*Hylexetastes perrotii*	9	5	3	2	5	3	2
	*Xenops minutus*	10	3	0	0	3	0	0
	*Hylocistes subulatus*	7	3	0	0	5	2	0
	*Automolus ochrolaemus*	9	3	1	0	2	1	0
	***Automolus infuscatus***	9	0	0	0	0	0	0
	*Philydor pyrrhodes*	9	2	0	0	2	0	0
	*Philydor erythrocercum*	9	3	3	1	3	3	1
High	***Xiphorhynchus picus***	7	0	0	0	0	0	0
	***Xiphorhynchus guttatus***	6	0	0	0	0	0	0
	*Lepidocolaptes albolineatus*	8	8	7	5	8	7	5
	*Xiphocolaptes promeropirhynchus*	8	2	1	0	2	1	0
	*Furnarius minor*	10	6	1	1	7	1	1
	*Furnarius leucopus*	10	7	6	3	7	6	3
	*Ancistrops strigilatus*	6	6	6	3	6	6	3
	*Philydor erythropterum*	9	2	1	0	2	1	0
	*Synallaxis albescens*	9	7	4	2	7	4	2
	*Synallaxis gujanensis*	9	4	2	0	4	2	0
	*Cranioleuca vulpina*	9	6	5	2	7	5	2
	Total dichromatic species		27	16	9	27	18	10

The greatest extent and magnitude of dichromatism were found in the high light group, in which 52.7% of the patches measured were dichromatic at the Δ*S* ≥ 1.0 threshold, with a mean jnd of 1.31 (Table 2). The low light group was second, with 37.8% of dichromatic patches, and an average jnd of 0.93. Lastly, the intermediate light group had 20.1% of dichromatic patches, and a mean jnd of 0.70. The analysis of variance with group as a block showed differences in jnd between the high and intermediate light groups, and the high and low light groups, but not between the intermediate and low light groups (post hoc Tukey test, *P*‐value < 0.05) (Table 2).

**Table 2:** Proportions of dichromatic patches (Δ*S* > 1.0) and mean discrimination values (in jnd units) by group, under the ideal Vorobyev–Osorio model of color discrimination. Letters show the results of post hoc tests (Tukey test) for each group, where groups with different letters are statistically different (*P*‐value < 0.05)

Light level	Total no. species	No. patches measured	% dichromatic patches (Δ*S* ≥ 1.0)	Discrimination (mean ± SD)
Low	4	37	37.8	0.93 ± 0.53^a^
Intermediate	18	144	20.1	0.70 ± 0.43^a^
High	11	91	52.7	1.31 ± 0.93^b^

**Figure 3:**
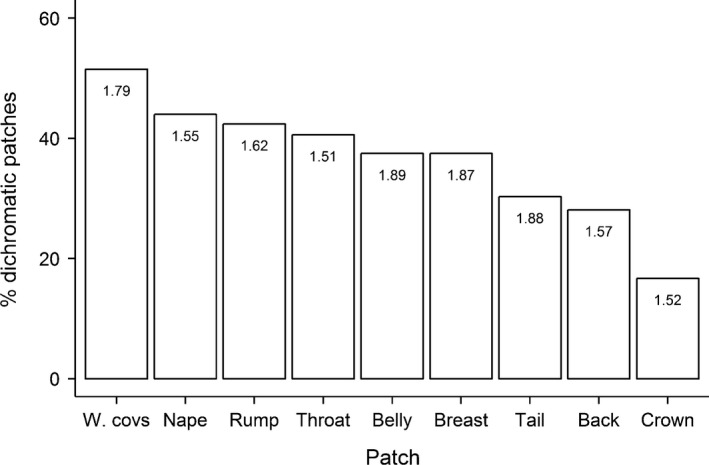
Proportion of dichromatic plumage patches by body region (Δ*S* > 1.0) identified by Vorobyev–Osorio model of color discrimination. Numbers in bars refer to the mean discrimination value (in jnd units) of dichromatic patches in each body region. All information obtained from the ideal model (see “Materials and Methods” for details of model parameters).

In all groups, the patches that were more frequently dichromatic were dorsal patches: wing coverts, nape, and rump, followed by ventral patches like throat and belly (Fig. 3) (Facial mark patches not included in this analysis). However, these patches did not have the highest discrimination values, as measured by their mean jnd (Fig. 3). Patches with the highest jnd scores were mostly ventral (breast and belly), as well as the tail. An analysis of variance with group as a block showed no differences among the jnd values for each patch (Table S1; *P*‐value = 0.66), yet groups were different among each other (*P*‐value < 0.05). Rerunning the models for each species with the ambient light spectra used for the other groups did not alter the results obtained (Table S2). The number of species having at least one dichromatic patch (Δ*S* ≥ 1.0) was the same under all the spectra considered, although the number of dichromatic patches identified varied for some species (Table S2).

### Interspecific variation

**Figure 4:**
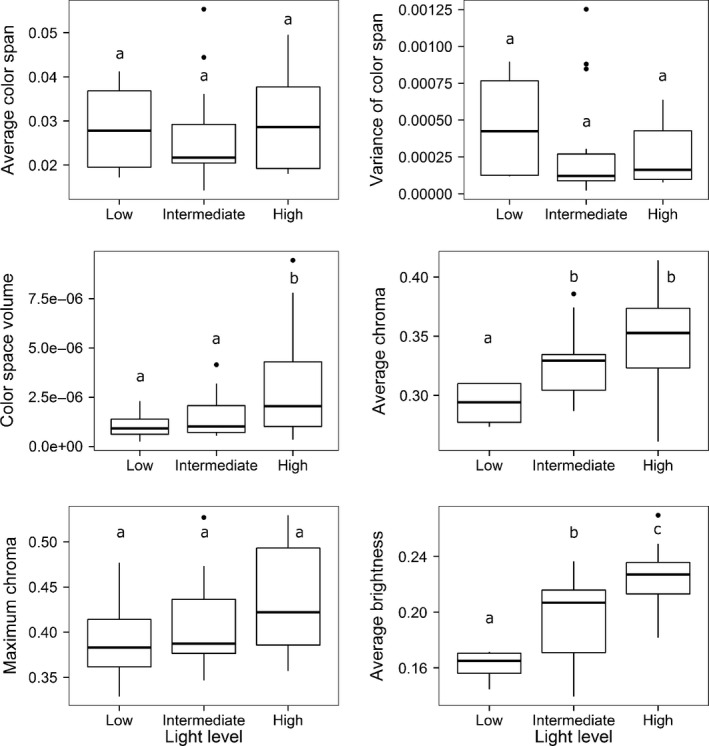
Whole‐plumage color characteristics and brightness of 33 bird species of the infraorder Furnariides, living in Amazonian habitats with different levels of ambient light. Letters show the results of post hoc tests (Tukey test) for each color descriptor, where groups with different letters are statistically different (*P*‐value < 0.05).

For each color descriptor, the analysis of variance with sex as a block showed no differences between males and females (*P*‐value > 0.05); therefore, results reported in this section are for both sexes of the same species combined. Summary statistics describing whole‐plumage color characteristics of each species are shown in Table 3. Color space volume, average chroma, and average brightness increased with increasing ambient light (Fig. 4). No significant differences were found for average color span, variance of color span, and maximum chroma (Fig. 4; *P*‐value > 0.05).

**Table 3:** Summary statistics describing whole‐plumage color characteristics and brightness of 33 bird species of the infraorder Furnariides, living in Amazonian habitats with different levels of ambient light

Species	Average color span	Variance of color span	Color space volume	Average chroma	Maximum chroma	Brightness
*F. analis*	0.02	1.18e‐4	7.50e‐7	0.31	0.37	0.17
*S. mexicanus*	0.04	8.97e‐4	1.10e‐6	0.31	0.48	0.14
*S. caudacutus*	0.02	1.28e‐4	2.50e‐7	0.28	0.33	0.16
*S. rutilans*	0.04	7.25e‐4	2.30e‐6	0.27	0.39	0.17
*D. merula*	0.02	4.66e‐5	8.00e‐7	0.33	0.37	0.14
*D. fuliginosa*	0.02	1.01e‐4	1.05e‐6	0.32	0.39	0.15
*D. stictolaema*	0.02	8.41e‐5	1.65e‐6	0.33	0.38	0.14
*D. longicauda*	0.01	2.21e‐5	7.00e‐7	0.37	0.39	0.15
*S. griseicapillus*	0.04	8.47e‐4	4.15e‐6	0.29	0.41	0.19
*G. spirurus*	0.03	2.72e‐4	2.20e‐6	0.33	0.44	0.16
*X. ocellatus*	0.02	8.36e‐5	6.50e‐7	0.30	0.36	0.21
*X. elegans*	0.02	8.93e‐5	5.50e‐7	0.34	0.41	0.19
*D. rufigula*	0.03	3.04e‐4	7.00e‐7	0.33	0.45	0.23
*D. certhia*	0.03	1.71e‐4	7.00e‐7	0.29	0.38	0.21
*D. picumnus*	0.02	9.80e‐5	1.10e‐6	0.32	0.38	0.21
*H. perrotii*	0.02	8.74e‐5	1.00e‐6	0.30	0.37	0.24
*X. minutus*	0.04	8.81e‐4	1.70e‐6	0.34	0.47	0.21
*H. subulatus*	0.02	9.36e‐5	7.50e‐7	0.33	0.38	0.19
*A. ochrolaemus*	0.03	1.77e‐4	3.15e‐6	0.37	0.46	0.22
*A. infuscatus*	0.02	1.41e‐4	8.50e‐7	0.31	0.35	0.22
*P. pyrrhodes*	0.06	1.25e‐3	2.25e‐6	0.39	0.53	0.21
*P. erythrocercum*	0.03	2.63e‐4	3.20e‐6	0.30	0.42	0.22
*X. picus*	0.02	8.34e‐5	6.50e‐7	0.31	0.38	0.22
*X. guttatus*	0.03	4.33e‐4	1.10e‐6	0.35	0.49	0.19
*L. albolineatus*	0.02	7.68e‐5	2.05e‐6	0.37	0.42	0.18
*X. promeropirhynchus*	0.02	1.42e‐4	3.50e‐7	0.26	0.36	0.23
*F. minor*	0.04	4.22e‐4	6.15e‐6	0.41	0.51	0.25
*F. leucopus*	0.05	6.38e‐4	9.45e‐6	0.39	0.50	0.27
*A. strigilatus*	0.03	1.62e‐4	2.45e‐6	0.34	0.40	0.24
*P. erythropterum*	0.04	5.75e‐4	7.80e‐6	0.37	0.53	0.23
*S. albescens*	0.02	9.64e‐5	9.50e‐7	0.33	0.39	0.22
*S. gujanensis*	0.02	1.00e‐4	2.05e‐6	0.31	0.36	0.21
*C. vulpina*	0.03	3.41e‐4	2.45e‐6	0.37	0.42	0.23

Average color span, a measure of contrast among color patches in a plumage, ranged from 0.01 (*Deconychura longicauda*) to 0.06 (*Philydor pyrrhodes*), both from the intermediate light (IL) group (Table 3). Range of variation in this variable is quite small because colors in the plumage of these species are basically shades of brown. Span variance is a measure of the uniformity of color contrast within a plumage. It ranged over two orders of magnitude, again from 2.21e‐5 in *D. longicauda* to 1.25e‐3 in *P. pyrrhodes* (Table 3). *Deconychura longicauda* is a very uniform bird, lacking the light throat patches of most Furnariides; therefore, its colors contrast with one another uniformly. On the other hand, *P. pyrrhodes* has a larger span variance because its color pattern is made up of two contrasting colors, which results in higher variance. Color space volume, a measure of color diversity, ranged over one order of magnitude, from 2.50e‐7 in *Sclerurus caudacutus* (low light group, LL) to 9.45e‐6 in *Furnarius leucopus* (high light group, HL) (Table 3). *Sclerurus caudacutus* is another bird which is very uniform in color, while *F. leucopus* has more diversity in colors, with strong white supercilium and throat, orangish upperparts, and a dark crown.

Average chroma, a measure of color saturation, varied from 0.26 (*Xiphocolaptes promeropirhynchus*) to 0.41 (*Furnarius minor*), both from the high light group. The throat patch was the most saturated patch in *X. promeropirhynchus*, scoring 0.36, while in *F. minor,* the breast patch was the highest in average chroma with a score of 0.51 (Table S3). Maximum chroma had a minimum of 0.33 in *S. caudacutus* (LL), and a maximum value of 0.53 in *P. pyrrhodes* (HL) and *P. erythropterum* (HL). Average brightness, the amount of light reflected by the plumage patch, ranged from 0.14 in *Sclerurus mexicanus* (LL), *Dendrocincla merula* (IL), and *Deconychura stictolaema* (IL) to 0.27 in *F. leucopus* (HL). The brightest patch in *S. mexicanus* and *D. merula* was the throat, with scores of 0.24 and 0.27, respectively. The brightest patch in *D. stictolaema* was the belly with a score of 0.18, while the brightest patch in *F. leucopus* was the throat with a score of 0.48 (Table S3).

### Evolution of plumage color

Support for all the three models of evolution was found depending on the color descriptor considered. Brightness fit an OU model with different selective regimes for ambient light considerably better than the other models (Table 4). This suggests that brightness evolution is constrained by the amount of ambient light available in the birds' habitats. The optimal value for low light level had the lowest score, followed by intermediate and high light groups, with no overlap in the 95% confidence intervals in the three light levels (Table 5). Variance of color span and maximum chroma fit an OU model with a single optimum better than the other models (*P*‐value < 0.05 for simpler models with AIC_c_ values of <4). Preference for the OU model with a single optimum in these two color characteristics is an indication of their evolution toward a single optimal value. Furthermore, all OU models had large *α* values, which adds support to strong stabilizing selection forces around optimal values (Table 5).

**Table 4:** Plumage evolution model comparison (ΔAIC_c_ values). Best‐fit model for each color descriptor is indicated in bold. A difference of <2 indicates no difference of fit in the more complex models

Color descriptor	Brownian motion	OU – single optimum	OU – light level
Average color span	**0.00**	0.35	8.05
Variance of color span	3.19*	**0.00**	7.05
Color space volume	**0.00**	9.45	11.76
Average chroma	**1.85**	0.21	0.00
Maximum chroma	3.63*	**0.00**	6.17
Average brightness	7.28	6.46	**0.00**

An asterisk “*” indicates a significant (*P*‐value < 0.05) likelihood ratio test between less‐complex models, and the less‐complex models can be rejected as a significantly worse fit to the data. A likelihood ratio test could not be used to compare models with a less‐complex model having the lower AIC_c_ value.

**Table 5:** Plumage model parameter estimates for the best‐fit model for each color descriptor (from Table 4). The most likely value is given, along with 95% confidence intervals calculated from 10,000 bootstrap replicates

Color descriptor	*α*	*σ* ^2^	Single optimum *θ*	Low *θ*	Intermediate *θ*	High *θ*
Average color span (BM)	–	6.1e‐6 (3.4e‐6, 9.3e‐6)	0.029 (0.01, 0.04)	–	–	–
Variance of color span (OU single)	3.71 (3.61, 4.62)	7.1e‐7 (4.4e‐7, 1.0e‐6)	3.0e‐4 (1.9e‐4, 4.4e‐4)	–	–	–
Color space volume (BM)	–	1.8e‐13 (1.0e‐13, 2.7e‐13)	2.0e‐6 (6.2e‐9, 4.1e‐6)	–	–	–
Average chroma (BM)	–	7.4e‐5 (4.1e‐5, 1.1e‐4)	0.32 (0.25, 0.38)	–	–	–
Maximum chroma (OU single)	4.61 (4.57, 5.66)	0.02 (0.01, 0.04)	0.41 (0.39, 0.43)	–	–	–
Average brightness (OU light level)	4.17 (4.07, 5.15)	0.005 (0.003, 0.008)	–	0.15 (0.12, 0.18)	0.19 (0.18, 0.20)	0.22 (0.20, 0.23)

Color space volume and average color span were best explained by a BM model (Table 4), which suggests that evolution of these color characteristics is unconstrained by ambient light levels. Lastly, average chroma best fits the most complex OU model (light level model), but I was not able to discriminate among the three models due to model selection uncertainty (ΔAIC_c_ < 2). Therefore, for this color descriptor, I chose BM as the best model.

## Discussion

Current understanding of the avian vision system and the phylogenetic relationships among birds opens new opportunities for exploring the evolution of plumage coloration in an avian‐appropriate perspective. I applied Vorobyev–Osorio model of color discrimination and the tetrahedral color space model, in combination with comparative methods to test the LEH in both intra‐ and interspecific contexts in a group of species of the infraorder Furnariides living under different ambient light regimes within the Amazon basin.

### LEH in the intraspecific context

Both the real and ideal models of color discrimination identified 27 species (81.8%) as having at least one dichromatic patch (Δ*S* > 1.0) within their plumage. However, numbers dropped significantly at the more conservative threshold (Δ*S* ≥ 2.0), where only 27.35% of the species were dichromatic under the real model. Overall, my results agree with previous studies exploring dichromatism in an avian visual perspective. For instance, Eaton (2005) found that 92.8% of 139 sexually monochromatic species from the order Passeriformes were dichromatic at the Δ*S* > 1.0 threshold. Burns and Shultz (2012) suggested that 97.3% of the cardinals and tanagers (376 species) are dichromatic at the same discrimination threshold, contrary to the 50% that were previously identified by human visual standards. Nonetheless, using the more conservative threshold, Eaton (2005) and Burns and Shultz (2012) estimated dichromatism levels of 60.4% and 76%, respectively, and maximum Δ*S* values > 10 jnd. In this study, the highest discrimination value was only 4.39 jnd in the belly of *F. minor* (HL) (Table S3).

In general, my results suggest that Furnariides reflect the same general pattern of sexual plumage dichromatism previously found in passerine birds, even though the magnitude of dichromatism does not seem to be as high as found in other groups. Considering that most of the species in this clade, and all of the species in this study, have been previously classified as sexually monochromatic by human standards, my results emphasize the importance of studying the evolution of sexual dichromatism from an avian visual perspective, as has been shown in previous studies (Eaton 2005; Burns and Shultz 2012). The use of human‐based scoring systems can produce very dissimilar results that can lead to misinterpretations of the evolutionary patterns of sexual dichromatism, with important implications in behavioral and ecological studies of birds (Eaton 2005; Burns and Shultz 2012).

Given that studies of dichromatism have mostly focused in quantifying the degree of divergence between sexes, it is hard to make comparisons under the ambient light approach. I found significant differences in the frequency and magnitude of dichromatism in species living under different light conditions. This finding supports my prediction of the role of the LEH in intraspecific variation, although in the opposite direction. On average, species from the high light group had more dichromatic patches and greater discrimination values than species from the intermediate and low light groups. This can be interpreted as enhanced communication in environments with high levels of ambient light, which was the opposite of what I predicted. Also, although not statistically significant, it was interesting to see that species from the intermediate light group had lower degrees of dichromatism than species from the low light group. Additionally, four of the six species identified as sexually monochromatic at all thresholds of discrimination were from this same group, creating a trend of decreased dichromatism in species from intermediate light habitats.

In all groups, the patches that were more frequently dichromatic were dorsal patches: wing coverts, nape, and rump, followed by ventral patches like throat and belly. This is interesting because of the characteristic foraging behavior of species of this clade, particularly woodcreepers, who spend most of their time climbing on tree trunks and rarely perch in an erect posture. Intraspecific variation in plumage color has been mostly related to communication, in which signalers reveal information about themselves to receivers (Hill and McGraw 2006b). Sexual differences have been attributed to recognition of sex‐related strategies and are particularly expected in species for which additional gender‐revealing cues are less apparent. Also, species whose genders have very similar roles during courtship and reproduction and those that are sexually monochromatic in appearance are predicted to have evolved sex recognition signals (Hill and McGraw 2006b). All species in the present study are sexually monochromatic based on human standards, and in most of them, both males and females develop brood patches, which suggest they both participate in egg incubation (E. Johnson, J.D. Wolfe, pers. obs.). Therefore, the fact that the patches that were more frequently dichromatic were from the dorsal area provides evidence of the role in sex recognition of these differences.

Lastly, one of my objectives was to quantify the extent of dichromatism under different levels of ambient light. Using Vorobyev–Osorio model of color discrimination with varying levels of ambient light (ideal and real models; see “Materials and Methods”), I found that, although the discrimination scores varied slightly, the conclusions drawn from them remained virtually unaffected. Similar results have been reported in previous studies (Eaton 2005; Stoddard and Prum 2008). For instance, Stoddard and Prum (2008) compared a simplified tetrahedral color space model to that of Endler and Mielke (2005) in two species of New World buntings under three different ambient light spectra and found that varying ambient light did not have a large effect on estimates of color perception. This is not surprising given that avian vision has evolved mechanisms for color constancy (Vorobyev et al. 2001; Stoddard and Prum 2008), but it is perhaps an invitation to increase the use of more pragmatic and simplified approaches which could represent a better option for studies of plumage color variation.

### LEH in the interspecific context

I found strong support for the LEH in the interspecific context, suggesting that ambient light plays an important role in shaping the plumage of this group of birds. Species signaling in the same light environments had similar color characteristics at least in terms of color diversity (color space volume), saturation (chroma), and brightness. Preference for OU models over the BM model in half of the color descriptors examined and the large *α* values for these models suggest strong directional selection for these plumage characteristics. Also, the fact that there were no differences between males and females suggests that both sexes may be under similar selective pressures.

Average brightness was the only trait to fit the more complex model that included different regimes based on the amount of ambient light available for each group. This suggests that ambient light is an important selective pressure for the evolution of plumage brightness, supporting previous observations (Endler 1993; McNaught and Owens 2002; Gomez and Thery 2004, 2007; Shultz and Burns 2013). Additionally, lower optimal values for this descriptor in the low light group and high values for the high light group serve as an indication of natural selection driving the evolution of cryptic color signals in these birds. As the forest understory receives considerably less light and has lower spatial light diversity than the canopy (Endler 1993), a bird with lower brightness in this type of habitat (or in generally darker habitats) will match the background in a more effective way than a brighter bird will do.

Support for decreased brightness in darker habitats has been found in other groups of birds. McNaught and Owens (2002) studied 65 species of Australian birds from six different families, living in closed and open environments, and found that birds from closed habitats used less bright colors than those used by birds from open habitats. Similarly, Gomez and Thery (2004) found that species from the understory of Tropical rainforests have developed less bright coloration than canopy species. Shultz and Burns (2013) also documented lower optimal brightness values in a clade of tanager species from the forest understory, suggesting that the evolution of plumage brightness is constrained by habitat characteristics.

Alternative explanations for the fact that birds with lower brightness were associated with low light habitats should also be considered. One possibility is that plumage signals are simply less effective in dark and dense vegetation and so plumage has tended to become duller because of selection against developmental costs, perhaps placing more emphasis on vocal communication. Structural plumage coloration is created by the coherent scattering of light caused by alternating layers of ordered keratin and air pockets within a feather's spongy medullary layer (Prum et al. 1999); thus, feather microstructure is thought to be produced with few costs (Prum 2006; but see Andersson 1999). Nonetheless, there is growing evidence that structural coloration is condition dependent and that it may serve as an honest signal of individual quality (Keyser and Hill 1999; Doucet 2002). Therefore, if ambient light is constraining visual communication in low light habitats, there is no evident need for the development of bright plumage under low light conditions. This idea goes in hand with my results of dichromatism. Contrary to my predictions, birds from the low and intermediate light groups had lower dichromatism than birds from the high light group. This gives reason to think that visual communication is more constrained in environments with low levels of ambient light. Whether by means of cryptic coloration by natural selection, or simply by constraining the efficiency of visual signaling in dark habitats, it appears that ambient light is an important factor limiting plumage brightness in these birds.

The best model for average chroma could not be identified due to model selection uncertainty. However, I did observe an increase in saturation with increasing ambient light. Endler's 4th rule of the interaction between ambient light and the reflectance of a patch states that the contrast of a color pattern in different light environments will be affected by the chroma of the component patches. The degree of saturation (chroma) of a patch determines the degree to which the patch appearance's will be affected by the color of ambient light (Endler 1993). As ambient light varies, unsaturated patches will vary more in color and brightness than saturated patches. As visual backgrounds consist mostly of low‐chroma patches, low‐chroma animals will contrast less than high‐chroma animals, but will also vary more with changing ambient light (Endler 1993).

Given the strong support for crypsis found in this and in previous studies, the decreased saturation in the plumage of birds from the low light group can be interpreted as another adaptation for crypsis. Endler (1978) predicted that when predation risk is high, cryptic color patterns should have patches with reflectance spectra similar to that of the background, as changes in appearance with ambient light may make unsaturated color patterns harder to recognize and track than saturated patterns (Endler 1993). On the other hand, given my results of dichromatism, that birds from the high light group had more saturated plumages can be explained as an adaptation for cryptic signaling. As mentioned earlier, canopy and open habitats have more variable ambient light; therefore, birds from these habitats will need more saturated plumages for constant appearance and easy recognition in any light environment (Endler 1993).

Even though color space volume best fit a Brownian motion model, which indicates random drift instead of directional selection, it also showed an increase with increasing ambient light. This color descriptor is a measure of color diversity, suggesting another adaptation for crypsis in this group of birds. As mentioned above, the canopy of forests has a higher spatial light diversity; therefore, increasing color diversity in the plumage pattern of these birds may result in increasing crypsis by matching the background of their habitat. Gomez and Thery (2004) reported similar results while studying a Neotropical rainforest bird community of 40 species. They classified species according to their foraging height, as either canopy or understory, and using comparative models they found that canopy birds had higher mean hue angles and more varied hues (their measure of color diversity) than ground birds, which they interpreted as an adaptation for crypsis. On the other hand, Shultz and Burns (2013) found the opposite in their study of plumage evolution in a clade of Neotropical tanagers. They found support for the evolution of color space volume as random drift (BM model over OU model), but only in females; males best fitted an OU model based on open versus closed habitats. However, they found higher optimal values of color space volume for the closed habitat group, which they interpreted as an adaptation for crypsis. Based on the argument that a closed environment with many different leaves, fruits, and flowers is more complex and has a greater diversity of colors, they suggested that a plumage pattern with high color diversity would be more cryptic in these kinds of environments.

Lastly, even though variance of color span and maximum chroma did not best fit an OU model with different selective regimes (no different optima for each ambient light level) and did not differ among the groups, they did fit the OU model with a single optimum better than the BM model. Parameters for these models indicate both strong selection forces around the optimal value (large *α*), and small drift for each color descriptor (small *σ* ^2^) (Butler and King 2004), which refutes stochastic processes as an alternative to nonadaptive color signals.

My results provide evidence for the role of ambient light in shaping plumage coloration in this group of birds at both intra‐ and interspecific levels in two main ways. First, the fact that birds from the high light group had higher levels of dichromatism, as well as brighter, more saturated and diverse plumages than birds from the other two groups suggests that ambient light constrains signal efficacy in low light environments. Interestingly, these same results also suggest that adaptation for crypsis prevails. Again, birds with higher brightness, saturation, and color diversity were associated with habitats with high levels of ambient light, which indicates that plumage in these birds has evolved to match the background in their respective habitats. Gomez and Thery (2007) found similar patterns of intra‐ and interspecific variation in bird species living in understory or canopy in a Tropical rainforest. Their highly detailed study helped reveal interesting patterns of crypsis and conspicuousness within a single plumage, helping understand how ambient light, in conjunction with natural and sexual selection, influences the evolution of plumage color in birds.

Tobias et al. (2010) found considerable support for acoustic adaptation in avian communities of two Amazonian forest types: bamboo and *terra firme*. Songs of birds from the two habitats differed in predictable ways according to the transmission properties of each environment, suggesting an important role of habitat structure in shaping the songs of these birds. This adds support to the view that physical characteristics of the environment influence how effectively signals are transmitted and received and that these signals and the associated sensory systems are adjusted to match characteristics of the environment. As these authors suggest, habitat heterogeneity, including ambient light levels, can cause divergent selection on signals associated with mate choice, potentially facilitating speciation, which could help explain the high levels of diversity in tropical birds.

Finally, for logistic reasons, I did not include all linages found in Amazonian habitats. However, in order to get an adequate representation of the whole range of variation, the species I included were selected from a variety of places within the clade. The evolution of plumage color signals in relation to ambient light appears to be a common pattern and has received significant support in single clades (Shultz and Burns 2013), as well as in unrelated groups who share similar habitats (McNaught and Owens 2002; Gomez and Thery 2004, 2007). It would be interesting to see whether the levels of dichromatism and the plumage patterns hold as more species are included, although given the range of variation already included in the present study, it is highly plausible that additional sampling might just reveal more detail into the pattern.

## Conclusions

My results suggest that ambient light plays a major role in the evolution of color signals in both intra‐ and interspecific contexts as well as support the idea that visual communication is constrained in environments with low levels of ambient light. Given that avian vision has evolved for advanced color discrimination and color constancy, it is expected that variation in ambient light spectral composition does not affect avian discriminatory capabilities to a great extent (Stoddard and Prum 2008). However, I have shown that the plumage of birds living in habitats with different levels of ambient light has different color characteristics: birds from the high light group were more likely to have higher dichromatism levels, as well as brighter, more saturated, and diverse plumages. My results also agree with the prediction that adaptation of color signals is driven by natural selection to enhance crypsis with the background. All of this goes in line with findings by previous studies, increasing support for the LEH as a possible explanation for interspecific variability in bird coloration, and in this particular case for intraspecific variation as well.

Analyzing dichromatism and color characteristics using both avian‐appropriate models and information about levels of ambient light available to transmit and receive the signals has helped to reveal interesting plumage patterns in this group of birds. As it has been shown here, it is important to use complementary models of avian color perception to strengthen analysis in this growing area of research (Kemp et al. 2015). The fact that adding ambient light spectra into models of avian vision did not have a significant effect on the results obtained suggests that this information is not very important. However, when accounting for the amount of light available in each bird's habitat, both dichromatism levels and plumage color characteristics varied in the predicted way. This is perhaps the most interesting result obtained here, as it highlights the importance of ambient light into shaping the plumage coloration of these drab birds, while also adds evidence for the little effect ambient light spectral data have in the models of avian vision themselves. There are likely some benefits to using models that make few assumptions, particularly when making comparisons among species. As more exciting and complex ecological and evolutionary questions about colors in nature arise, there is an increasing need for simple, straightforward methods that allow comparisons within and among species and studies.

## Conflict of Interest

The author declares that there is no conflict of interest.

## Supporting information


**Table S1.** Proportion of dichromatic plumage patches (Δ*S* > 1.0) and mean discrimination values (in jnd units) by body region, under the ideal Vorobyev–Osorio model of color discrimination.
**Table S2.** Number of dichromatic plumage patches (Δ*S* ≥ 1.0) obtained using the Vorobyev–Osorio model of color discrimination for 33 bird species of the infraorder Furnariides living in Amazonian habitats with different levels of ambient light.
**Table S3.** Descriptions of color and brightness of all plumage patches measured from males and females of 33 bird species of the infraorder Furnariides, living in Amazonian habitats with different levels of ambient light.
