# Supplementary material for: Light matters: testing the “Light Environment Hypothesis” under intra‐ and interspecific contexts
Source: Ecol Evol. 2016 May 18;6(12):4018–31. doi: 10.1002/ece3.2188 (PMC4875815; doi:10.1002/ece3.2188)
Supplement: Supplementary file 1 — Table S1. Proportion of dichromatic plumage patches (ΔS > 1.0) and mean discrimination values (in jnd units) by body region, under the ideal Vorobyev–Osorio model of color discrimination. Table S2. Number of dichromatic plumage patches (ΔS ≥ 1.0) obtained using the Vorobyev–Osorio model of color discrimination for 33 bird species of the infraorder Furnariides living in Amazonian habitats with different levels of ambient light. Table S3. Descriptions of color and brightness of all plumage patches measured from males and females of 33 bird species of the infraorder Furnariides, living in Amazonian habitats with different levels of ambient light. [file ECE3-6-4018-s001.docx]

**Table S1.** Proportion of dichromatic plumage patches (ΔS > 1.0) and mean discrimination values (in jnd units) by body region, under the ideal Vorobyev-Osorio model of color discrimination. n refers to the number of species in which each patch was measured. Letters show the results of post-hoc tests (Tukey test), where patches with different letters are statistically different (p-value<0.05).

| **Patch** | **n** | **Frequency** | **Proportion (%)** | **Discrimination (mean ± sd)** |
| --- | --- | --- | --- | --- |
| Facial mark | 4 | 3 | 75.0 | 2.12 ± 1.15 ^a^ |
| Belly | 32 | 12 | 37.5 | 1.89 ± 0.93 ^a^ |
| Tail | 33 | 10 | 30.3 | 1.88 ± 0.93 ^a^ |
| Breast | 24 | 9 | 37.5 | 1.87 ± 0.90 ^a^ |
| Wing Covs | 33 | 17 | 51.5 | 1.79 ± 0.62 ^a^ |
| Rump | 33 | 14 | 42.4 | 1.62 ± 0.54 ^a^ |
| Back | 32 | 9 | 28.1 | 1.57 ± 0.51 ^a^ |
| Nape | 25 | 11 | 44.0 | 1.55 ± 0.35 ^a^ |
| Crown | 24 | 4 | 16.7 | 1.52 ± 0.59 ^a^ |
| Throat | 32 | 13 | 40.6 | 1.51 ± 0.51 ^a^ |

**Table S2.** Number of dichromatic plumage patches (S ≥ 1.0) obtained using the Vorobyev-Osorio model of color discrimination for 33 bird species of the infraorder Furnariides living in Amazonian habitats with different levels of ambient light. Four different ambient light spectra were evaluated for each species (ideal, forest shade, standard daylight, and blue sky). Species in bold were identified as completely monochromatic under all different spectra.

| **Light level** | **Species** | **# patches measured** | **Number of dichromatic patches** | | | |
| --- | --- | --- | --- | --- | --- | --- |
|  |  |  | **Ideal** | **Forest shade** | **Standard daylight** | **Blue sky** |
| Low | *Formicarius analis* | 10 | 6 | 6 | 5 | 6 |
|  | *Sclerurus mexicanus* | 9 | 7 | 6 | 4 | 7 |
|  | *Sclerurus caudacutus* | 9 | 1 | 1 | 1 | 1 |
|  | *Synallaxis rutilans* | 9 | 1 | 1 | 1 | 1 |
| Intermediate | *Dendrocincla merula* | 9 | 1 | 1 | 1 | 1 |
|  | *Dendrocincla fuliginosa* | 9 | 3 | 2 | 1 | 3 |
|  | *Deconychura stictolaema* | 6 | 4 | 2 | 2 | 4 |
|  | *Deconychura longicauda* | 6 | 1 | 1 | 1 | 1 |
|  | *Sittasomus griseicapillus* | 9 | 2 | 2 | 2 | 2 |
|  | ***Glyphorynchus spirurus*** | 8 | 0 | 0 | 0 | 0 |
|  | *Xiphorhynchus ocellatus* | 6 | 3 | 2 | 1 | 3 |
|  | *Xiphorhynchus elegans* | 6 | 2 | 2 | 1 | 2 |
|  | ***Dendrexetastes rufigula*** | 9 | 0 | 0 | 0 | 0 |
|  | ***Dendrocolaptes certhia*** | 5 | 0 | 0 | 0 | 0 |
|  | *Dendrocolaptes picumnus* | 9 | 1 | 1 | 1 | 1 |
|  | *Hylexetastes perrotii* | 9 | 5 | 5 | 5 | 5 |
|  | *Xenops minutus* | 10 | 3 | 3 | 3 | 3 |
|  | *Hylocistes subulatus* | 7 | 5 | 5 | 3 | 5 |
|  | *Automolus ochrolaemus* | 9 | 2 | 2 | 3 | 2 |
|  | ***Automolus infuscatus*** | 9 | 0 | 0 | 0 | 0 |
|  | *Philydor pyrrhodes* | 9 | 2 | 2 | 2 | 2 |
|  | *Philydor erythrocercum* | 9 | 3 | 3 | 3 | 3 |
| High | ***Xiphorhynchus picus*** | 7 | 0 | 0 | 0 | 0 |
|  | ***Xiphorhynchus guttatus*** | 6 | 0 | 0 | 0 | 0 |
|  | *Lepidocolaptes albolineatus* | 8 | 8 | 8 | 8 | 8 |
|  | *Xiphocolaptes promeropirhynchus* | 8 | 2 | 1 | 1 | 2 |
|  | *Furnarius minor* | 10 | 7 | 5 | 5 | 6 |
|  | *Furnarius leucopus* | 10 | 7 | 7 | 7 | 7 |
|  | *Ancistrops strigilatus* | 6 | 6 | 6 | 6 | 6 |
|  | *Philydor erythropterum* | 9 | 2 | 2 | 1 | 2 |
|  | *Synallaxis albescens* | 9 | 7 | 7 | 7 | 7 |
|  | *Synallaxis gujanensis* | 9 | 4 | 4 | 4 | 4 |
|  | *Cranioleuca vulpina* | 9 | 7 | 6 | 6 | 7 |
|  | Total dichromatic species |  | 27 | 27 | 27 | 27 |
|  | Total dichromatic patches |  | 102 | 93 | 85 | 101 |

**Table S3.** Descriptions of color and brightness of all plumage patches measured from males and females of 33 bird species of the infraorder Furnariides, living in Amazonian habitats with different levels of ambient light. Units in parenthesis. ΔS represents distance in avian color space between males and females in each body region, is expressed in “just noticeable differences” (jnd), where 1.0 is the threshold value for discrimination of two colors by birds.

| **Patch** | **Average chroma** | | **Maximum chroma** | | **Brightness (%)** | | **Ideal model ΔS (jnd)** | | **Real**  **model ΔS**  **(jnd)** | |
| --- | --- | --- | --- | --- | --- | --- | --- | --- | --- | --- |
| *F. analis* |  | |  | |  | |  | |  | |
| Throat | 0.23 | | 0.28 | | 12.35 | | 0.83 | | 0.60 | |
| Breast | 0.30 | | 0.28 | | 23.00 | | 1.95 | | 1.64 | |
| Belly | 0.37 | | 0.29 | | 27.50 | | 0.57 | | 0.42 | |
| Crown | 0.31 | | 0.29 | | 13.59 | | 0.87 | | 0.75 | |
| Nape | 0.31 | | 0.31 | | 14.54 | | 1.91 | | 1.66 | |
| Back | 0.34 | | 0.32 | | 14.53 | | 1.41 | | 1.06 | |
| Rump | 0.34 | | 0.31 | | 15.10 | | 0.99 | | 0.70 | |
| Tail | 0.28 | | 0.30 | | 13.72 | | 1.10 | | 1.03 | |
| Wing Covs | 0.34 | | 0.31 | | 14.26 | | 1.91 | | 1.56 | |
| Facial mark | 0.30 | | 0.28 | | 25.49 | | 2.05 | | 1.89 | |
| *S. mexicanus* |  | |  | |  | |  | |  | |
| Throat | 0.48 | | 0.36 | | 24.90 | | 2.82 | | 2.78 | |
| Breast | 0.36 | | 0.34 | | 15.48 | | 1.32 | | 1.14 | |
| Belly | 0.32 | | 0.31 | | 14.07 | | 0.50 | | 0.34 | |
| Crown | 0.26 | | 0.29 | | 12.75 | | 0.84 | | 0.64 | |
| Nape | 0.28 | | 0.30 | | 14.27 | | 1.33 | | 1.09 | |
| Back | 0.30 | | 0.31 | | 13.07 | | 1.03 | | 0.81 | |
| Rump | 0.30 | | 0.32 | | 13.75 | | 1.69 | | 1.53 | |
| Tail | 0.24 | | 0.28 | | 11.94 | | 1.56 | | 1.25 | |
| Wing Covs | 0.31 | | 0.31 | | 12.92 | | 1.57 | | 1.20 | |
| *S. caudacutus* |  | |  | |  | |  | |  | |
| Throat | 0.30 | | 0.29 | | 32.26 | | 0.84 | | 0.81 | |
| Breast | 0.33 | | 0.33 | | 15.68 | | 0.85 | | 0.80 | |
| Belly | 0.30 | | 0.31 | | 14.65 | | 1.77 | | 1.64 | |
| Crown | 0.28 | | 0.32 | | 13.92 | | 0.66 | | 0.63 | |
| Nape | 0.29 | | 0.32 | | 14.52 | | 0.49 | | 0.49 | |
| Back | 0.28 | | 0.32 | | 13.99 | | 0.42 | | 0.33 | |
| Rump | 0.29 | | 0.32 | | 14.38 | | 0.96 | | 0.83 | |
| Tail | 0.21 | | 0.30 | | 13.35 | | 0.89 | | 0.83 | |
| Wing Covs | 0.27 | | 0.32 | | 14.49 | | 0.90 | | 0.86 | |
| *S. rutilans* |  | |  | |  | |  | |  | |
| Throat | 0.18 | | 0.29 | | 14.88 | | 0.34 | | 0.27 | |
| Breast | 0.33 | | 0.36 | | 18.29 | | 0.60 | | 0.58 | |
| Belly | 0.31 | | 0.31 | | 19.48 | | 0.52 | | 0.52 | |
| Crown | 0.39 | | 0.38 | | 22.92 | | 1.22 | | 1.20 | |
| Nape | 0.28 | | 0.31 | | 15.99 | | 0.34 | | 0.34 | |
| Back | 0.30 | | 0.32 | | 16.06 | | 0.59 | | 0.46 | |
| Rump | 0.26 | | 0.30 | | 16.04 | | 0.95 | | 0.84 | |
| Tail | 0.17 | | 0.28 | | 15.54 | | 0.21 | | 0.18 | |
| Wing Covs | 0.30 | | 0.33 | | 15.59 | | 0.87 | | 0.75 | |
| *D. merula* |  | |  | |  | |  | |  | |
| Throat | 0.30 | | 0.32 | | 27.65 | | 1.21 | | 1.04 | |
| Breast | 0.33 | | 0.33 | | 13.93 | | 0.48 | | 0.49 | |
| Belly | 0.37 | | 0.34 | | 14.99 | | 0.86 | | 0.87 | |
| Crown | 0.31 | | 0.32 | | 11.80 | | 0.84 | | 0.75 | |
| Nape | 0.33 | | 0.33 | | 12.76 | | 0.26 | | 0.18 | |
| Back | 0.34 | | 0.33 | | 13.10 | | 0.36 | | 0.39 | |
| Rump | 0.35 | | 0.34 | | 13.58 | | 0.24 | | 0.21 | |
| Tail | 0.32 | | 0.34 | | 11.67 | | 0.34 | | 0.27 | |
| Wing Covs | 0.33 | | 0.34 | | 12.47 | | 0.38 | | 0.25 | |
| *D. fuliginosa* |  | |  | |  | |  | |  | |
| Throat | 0.32 | | 0.31 | | 24.19 | | 0.56 | | 0.56 | |
| Breast | 0.36 | | 0.34 | | 16.52 | | 0.53 | | 0.54 | |
| Belly | 0.39 | | 0.34 | | 16.73 | | 1.15 | | 1.11 | |
| Crown | 0.30 | | 0.32 | | 14.10 | | 0.82 | | 0.72 | |
| Nape | 0.29 | | 0.32 | | 14.00 | | 1.04 | | 0.97 | |
| Back | 0.32 | | 0.33 | | 13.03 | | 0.99 | | 0.90 | |
| Rump | 0.34 | | 0.34 | | 14.40 | | 0.50 | | 0.40 | |
| Tail | 0.27 | | 0.35 | | 13.26 | | 0.97 | | 0.86 | |
| Wing Covs | 0.29 | | 0.33 | | 13.19 | | 1.17 | | 0.90 | |
| *D. stictolaema* |  | |  | |  | |  | |  | |
| Belly | 0.38 | | 0.32 | | 18.67 | | 1.17 | | 1.02 | |
| Nape | 0.28 | | 0.31 | | 13.13 | | 1.06 | | 0.81 | |
| Back | 0.32 | | 0.33 | | 14.02 | | 0.88 | | 0.70 | |
| Rump | 0.35 | | 0.35 | | 13.93 | | 0.59 | | 0.48 | |
| Tail | 0.30 | | 0.33 | | 12.32 | | 1.84 | | 1.24 | |
| Wing Covs | 0.34 | | 0.32 | | 13.26 | | 1.19 | | 0.78 | |
| *D. longicauda* |  | |  | |  | |  | |  | |
| Throat | 0.37 | | 0.31 | | 20.68 | | 0.42 | | 0.42 | |
| Belly | 0.39 | | 0.32 | | 17.04 | | 0.14 | | 0.20 | |
| Back | 0.35 | | 0.32 | | 13.79 | | 0.74 | | 0.64 | |
| Rump | 0.37 | | 0.33 | | 13.81 | | 1.18 | | 1.16 | |
| Tail | 0.35 | | 0.32 | | 11.57 | | 0.87 | | 0.77 | |
| Wing Covs | 0.38 | | 0.31 | | 13.44 | | 0.78 | | 0.67 | |
| *S. griseicapillus* |  | |  | |  | |  | |  | |
| Throat | 0.23 | | 0.35 | | 23.91 | | 0.44 | | 0.36 | |
| Breast | 0.26 | | 0.34 | | 22.00 | | 0.84 | | 0.89 | |
| Belly | 0.27 | | 0.34 | | 26.17 | | 1.43 | | 1.47 | |
| Crown | 0.22 | | 0.33 | | 16.15 | | 0.20 | | 0.27 | |
| Nape | 0.19 | | 0.34 | | 16.66 | | 0.42 | | 0.51 | |
| Back | 0.28 | | 0.34 | | 17.56 | | 0.38 | | 0.35 | |
| Rump | 0.41 | | 0.39 | | 19.67 | | 1.18 | | 1.15 | |
| Tail | 0.36 | | 0.39 | | 15.95 | | 0.45 | | 0.43 | |
| Wing Covs | 0.34 | | 0.35 | | 16.75 | | 0.98 | | 0.88 | |
| *G. spirurus* |  | |  | |  | |  | |  | |
| Throat | 0.44 | | 0.36 | | 24.75 | | 0.70 | | 0.70 | |
| Belly | 0.34 | | 0.33 | | 19.79 | | 0.14 | | 0.10 | |
| Crown | 0.28 | | 0.32 | | 14.38 | | 0.56 | | 0.56 | |
| Nape | 0.31 | | 0.34 | | 14.77 | | 0.24 | | 0.21 | |
| Back | 0.34 | | 0.36 | | 15.76 | | 0.16 | | 0.17 | |
| Rump | 0.37 | | 0.38 | | 16.51 | | 0.38 | | 0.29 | |
| Tail | 0.29 | | 0.36 | | 13.90 | | 0.57 | | 0.49 | |
| Wing Covs | 0.31 | | 0.34 | | 14.76 | | 0.78 | | 0.71 | |
| *X. ocellatus* |  | |  | |  | |  | |  | |
| Throat | 0.36 | | 0.32 | | 29.30 | | 1.59 | | 1.65 | |
| Belly | 0.33 | | 0.33 | | 24.52 | | 0.59 | | 0.62 | |
| Back | 0.29 | | 0.32 | | 17.93 | | 0.81 | | 0.45 | |
| Rump | 0.29 | | 0.36 | | 19.16 | | 1.10 | | 0.92 | |
| Tail | 0.24 | | 0.35 | | 18.59 | | 0.86 | | 0.56 | |
| Wing Covs | 0.29 | | 0.32 | | 18.21 | | 1.51 | | 0.98 | |
| *X. elegans* |  | |  | |  | |  | |  | |
| Throat | 0.41 | | 0.32 | | 26.53 | | 1.04 | | 0.99 | |
| Belly | 0.39 | | 0.32 | | 23.53 | | 1.13 | | 1.07 | |
| Back | 0.32 | | 0.32 | | 16.38 | | 0.96 | | 0.97 | |
| Rump | 0.35 | | 0.35 | | 16.94 | | 0.69 | | 0.67 | |
| Tail | 0.28 | | 0.34 | | 16.44 | | 0.67 | | 0.49 | |
| Wing Covs | 0.34 | | 0.32 | | 16.99 | | 0.35 | | 0.37 | |
| *D. rufigula* |  | |  | |  | |  | |  | |
| Throat | 0.45 | | 0.34 | | 30.21 | | 0.78 | | 0.67 | |
| Breast | 0.36 | | 0.33 | | 24.44 | | 0.45 | | 0.35 | |
| Belly | 0.40 | | 0.34 | | 26.35 | | 0.26 | | 0.26 | |
| Crown | 0.33 | | 0.31 | | 22.88 | | 0.57 | | 0.53 | |
| Nape | 0.32 | | 0.31 | | 22.81 | | 0.37 | | 0.33 | |
| Back | 0.31 | | 0.32 | | 20.20 | | 0.55 | | 0.44 | |
| Rump | 0.33 | | 0.33 | | 21.38 | | 0.74 | | 0.73 | |
| Tail | 0.26 | | 0.33 | | 19.29 | | 0.60 | | 0.42 | |
| Wing Covs | 0.30 | | 0.32 | | 19.91 | | 0.49 | | 0.39 | |
| *D. certhia* |  | |  | |  | |  | |  | |
| Throat | 0.29 | | 0.30 | | 28.70 | | 0.94 | | 0.92 | |
| Belly | 0.38 | | 0.34 | | 24.56 | | 0.31 | | 0.31 | |
| Rump | 0.30 | | 0.35 | | 19.46 | | 0.28 | | 0.24 | |
| Tail | 0.23 | | 0.34 | | 17.98 | | 0.53 | | 0.45 | |
| Wing Covs | 0.29 | | 0.33 | | 18.85 | | 0.41 | | 0.34 | |
| *D. picumnus* |  | |  | |  | |  | |  | |
| Throat | 0.37 | | 0.32 | | 27.29 | | 0.64 | | 0.64 | |
| Breast | 0.36 | | 0.33 | | 22.08 | | 0.65 | | 0.67 | |
| Belly | 0.38 | | 0.34 | | 22.64 | | 0.10 | | 0.12 | |
| Crown | 0.30 | | 0.31 | | 19.10 | | 0.34 | | 0.35 | |
| Nape | 0.31 | | 0.32 | | 19.95 | | 1.39 | | 1.38 | |
| Back | 0.33 | | 0.33 | | 19.39 | | 0.46 | | 0.49 | |
| Rump | 0.32 | | 0.35 | | 19.01 | | 0.30 | | 0.31 | |
| Tail | 0.25 | | 0.34 | | 18.01 | | 0.91 | | 0.93 | |
| Wing Covs | 0.32 | | 0.33 | | 19.19 | | 0.58 | | 0.40 | |
| *H. perrotii* |  | |  | |  | |  | |  | |
| Throat | 0.32 | | 0.31 | | 30.50 | | 1.42 | | 1.22 | |
| Breast | 0.33 | | 0.31 | | 25.41 | | 1.09 | | 1.12 | |
| Belly | 0.37 | | 0.32 | | 32.43 | | 0.76 | | 0.52 | |
| Crown | 0.29 | | 0.31 | | 21.06 | | 0.63 | | 0.63 | |
| Nape | 0.29 | | 0.31 | | 23.09 | | 0.76 | | 0.69 | |
| Back | 0.31 | | 0.33 | | 20.16 | | 1.58 | | 1.60 | |
| Rump | 0.32 | | 0.36 | | 22.14 | | 2.65 | | 2.73 | |
| Tail | 0.25 | | 0.34 | | 19.44 | | 0.82 | | 0.80 | |
| Wing Covs | 0.29 | | 0.33 | | 21.15 | | 2.28 | | 2.29 | |
| *X. minutus* |  | |  | |  | |  | |  | |
| Throat | 0.29 | | 0.30 | | 33.20 | | 0.53 | | 0.52 | |
| Breast | 0.33 | | 0.32 | | 19.73 | | 0.22 | | 0.18 | |
| Belly | 0.33 | | 0.32 | | 21.07 | | 1.34 | | 1.35 | |
| Crown | 0.32 | | 0.31 | | 14.81 | | 0.55 | | 0.55 | |
| Nape | 0.32 | | 0.32 | | 15.00 | | 0.42 | | 0.29 | |
| Back | 0.32 | | 0.32 | | 15.63 | | 0.49 | | 0.38 | |
| Rump | 0.34 | | 0.33 | | 16.39 | | 1.05 | | 1.08 | |
| Tail | 0.44 | | 0.36 | | 18.89 | | 0.92 | | 0.94 | |
| Wing Covs | 0.30 | | 0.31 | | 14.47 | | 1.23 | | 1.18 | |
| Facial mark | 0.22 | | 0.27 | | 45.30 | | 0.56 | | 0.59 | |
| *H. subulatus* |  | |  | |  | |  | |  | |
| Throat | 0.37 | | 0.33 | | 27.80 | | 0.75 | | 0.65 | |
| Breast | 0.36 | | 0.33 | | 22.92 | | 1.04 | | 1.09 | |
| Belly | 0.38 | | 0.34 | | 22.93 | | 0.89 | | 0.92 | |
| Back | 0.30 | | 0.31 | | 15.66 | | 1.32 | | 0.84 | |
| Rump | 0.33 | | 0.34 | | 16.78 | | 1.39 | | 1.06 | |
| Tail | 0.29 | | 0.34 | | 16.19 | | 1.90 | | 1.15 | |
| Wing Covs | 0.31 | | 0.32 | | 16.00 | | 1.62 | | 0.97 | |
| *A. ochrolaemus* |  | |  | |  | |  | |  | |
| Throat | 0.42 | | 0.30 | | 44.48 | | 0.49 | | 0.41 | |
| Breast | 0.46 | | 0.32 | | 32.22 | | 0.31 | | 0.19 | |
| Belly | 0.43 | | 0.32 | | 30.23 | | 0.46 | | 0.51 | |
| Crown | 0.32 | | 0.31 | | 17.35 | | 0.71 | | 0.77 | |
| Nape | 0.31 | | 0.30 | | 15.56 | | 0.94 | | 1.01 | |
| Back | 0.32 | | 0.31 | | 16.20 | | 0.30 | | 0.31 | |
| Rump | 0.39 | | 0.36 | | 18.59 | | 0.54 | | 0.55 | |
| Tail | 0.36 | | 0.35 | | 15.76 | | 1.70 | | 1.64 | |
| Wing Covs | 0.35 | | 0.32 | | 16.19 | | 1.09 | | 1.07 | |
| *A. infuscatus* |  | |  | |  | |  | |  | |
| Throat | 0.30 | | 0.30 | | 45.97 | | 0.48 | | 0.48 | |
| Breast | 0.33 | | 0.31 | | 34.03 | | 0.68 | | 0.67 | |
| Belly | 0.34 | | 0.32 | | 31.12 | | 0.38 | | 0.37 | |
| Crown | 0.29 | | 0.31 | | 15.50 | | 0.74 | | 0.64 | |
| Nape | 0.28 | | 0.30 | | 14.42 | | 0.57 | | 0.46 | |
| Back | 0.29 | | 0.31 | | 15.30 | | 0.59 | | 0.52 | |
| Rump | 0.34 | | 0.35 | | 16.21 | | 0.24 | | 0.27 | |
| Tail | 0.31 | | 0.35 | | 14.75 | | 0.71 | | 0.74 | |
| Wing Covs | 0.29 | | 0.32 | | 15.39 | | 0.58 | | 0.60 | |
| *P. pyrrhodes* |  | |  | |  | |  | |  | |
| Throat | 0.53 | | 0.43 | | 27.83 | | 1.44 | | 1.45 | |
| Breast | 0.48 | | 0.41 | | 24.57 | | 0.27 | | 0.27 | |
| Belly | 0.47 | | 0.41 | | 24.20 | | 0.33 | | 0.31 | |
| Crown | 0.29 | | 0.31 | | 16.42 | | 0.06 | | 0.09 | |
| Nape | 0.32 | | 0.33 | | 17.41 | | 0.48 | | 0.43 | |
| Back | 0.33 | | 0.33 | | 16.99 | | 0.37 | | 0.30 | |
| Rump | 0.44 | | 0.40 | | 21.68 | | 0.51 | | 0.51 | |
| Tail | 0.39 | | 0.40 | | 22.35 | | 1.05 | | 1.05 | |
| Wing Covs | 0.21 | | 0.27 | | 15.84 | | 0.18 | | 0.16 | |
| *P. erythrocercum* |  | |  | |  | |  | |  | |
| Throat | 0.42 | | 0.34 | | 36.37 | | 0.77 | | 0.85 | |
| Breast | 0.34 | | 0.34 | | 30.32 | | 1.76 | | 1.72 | |
| Belly | 0.32 | | 0.33 | | 26.56 | | 1.55 | | 1.58 | |
| Crown | 0.25 | | 0.32 | | 16.54 | | 0.37 | | 0.27 | |
| Nape | 0.23 | | 0.33 | | 17.77 | | 2.08 | | 2.07 | |
| Back | 0.28 | | 0.33 | | 16.79 | | 0.59 | | 0.37 | |
| Rump | 0.30 | | 0.35 | | 18.04 | | 0.80 | | 0.75 | |
| Tail | 0.29 | | 0.39 | | 20.26 | | 0.61 | | 0.44 | |
| Wing Covs | 0.26 | | 0.33 | | 17.50 | | 0.63 | | 0.56 | |
| *X. picus* |  | |  | |  | |  | |  | |
| Throat | 0.38 | | 0.31 | | 39.40 | | 0.67 | | 0.65 | |
| Breast | 0.33 | | 0.32 | | 21.48 | | 0.51 | | 0.51 | |
| Belly | 0.35 | | 0.33 | | 22.08 | | 0.37 | | 0.35 | |
| Back | 0.32 | | 0.34 | | 18.87 | | 0.29 | | 0.29 | |
| Rump | 0.32 | | 0.35 | | 19.40 | | 0.72 | | 0.73 | |
| Tail | 0.26 | | 0.33 | | 18.44 | | 0.98 | | 0.97 | |
| Wing Covs | 0.28 | | 0.33 | | 19.18 | | 0.59 | | 0.57 | |
| *X. guttatus* |  | |  | |  | |  | |  | |
| Throat | 0.49 | | 0.33 | | 32.01 | | 0.28 | | 0.28 | |
| Belly | 0.41 | | 0.34 | | 20.74 | | 0.36 | | 0.38 | |
| Back | 0.34 | | 0.32 | | 15.42 | | 0.48 | | 0.48 | |
| Rump | 0.34 | | 0.34 | | 15.82 | | 0.53 | | 0.51 | |
| Tail | 0.27 | | 0.33 | | 16.20 | | 0.54 | | 0.52 | |
| Wing Covs | 0.34 | | 0.32 | | 15.83 | | 0.61 | | 0.59 | |
| *L. albolineatus* |  | |  | |  | |  | |  | |
| Throat | 0.40 | | 0.30 | | 31.47 | | 1.09 | | 1.07 | |
| Belly | 0.37 | | 0.30 | | 22.82 | | 1.66 | | 1.65 | |
| Crown | 0.33 | | 0.30 | | 15.94 | | 2.39 | | 2.38 | |
| Nape | 0.35 | | 0.31 | | 16.22 | | 1.92 | | 1.92 | |
| Back | 0.37 | | 0.32 | | 16.72 | | 2.36 | | 2.35 | |
| Rump | 0.38 | | 0.35 | | 17.39 | | 2.21 | | 2.20 | |
| Tail | 0.34 | | 0.33 | | 13.90 | | 3.81 | | 3.76 | |
| Wing Covs | 0.37 | | 0.31 | | 16.28 | | 3.18 | | 3.16 | |
| *X. promeropirhynchus* | |  | |  | |  | |  | |  |
| Throat | 0.36 | | 0.32 | | 31.59 | | 1.03 | | 1.01 | |
| Breast | 0.28 | | 0.32 | | 23.68 | | 0.30 | | 0.29 | |
| Crown | 0.22 | | 0.31 | | 21.50 | | 0.41 | | 0.39 | |
| Nape | 0.25 | | 0.31 | | 22.34 | | 1.69 | | 1.66 | |
| Back | 0.27 | | 0.32 | | 21.09 | | 0.34 | | 0.32 | |
| Rump | 0.27 | | 0.33 | | 21.21 | | 0.58 | | 0.59 | |
| Tail | 0.21 | | 0.33 | | 20.88 | | 0.35 | | 0.32 | |
| Wing Covs | 0.25 | | 0.33 | | 22.36 | | 0.23 | | 0.23 | |
| *F. minor* |  | |  | |  | |  | |  | |
| Throat | 0.37 | | 0.28 | | 41.35 | | 1.02 | | 1.02 | |
| Breast | 0.51 | | 0.31 | | 30.23 | | 0.36 | | 0.35 | |
| Belly | 0.44 | | 0.28 | | 46.02 | | 4.39 | | 4.37 | |
| Crown | 0.33 | | 0.31 | | 17.50 | | 1.35 | | 1.32 | |
| Nape | 0.35 | | 0.31 | | 17.31 | | 0.90 | | 0.89 | |
| Back | 0.45 | | 0.37 | | 19.21 | | 0.94 | | 0.92 | |
| Rump | 0.49 | | 0.36 | | 21.66 | | 1.13 | | 1.12 | |
| Tail | 0.37 | | 0.36 | | 15.43 | | 1.27 | | 1.26 | |
| Wing Covs | 0.42 | | 0.36 | | 17.90 | | 1.49 | | 1.47 | |
| Facial mark | 0.45 | | 0.29 | | 32.00 | | 1.01 | | 0.99 | |
| *F. leucopus* |  | |  | |  | |  | |  | |
| Throat | 0.31 | | 0.27 | | 48.56 | | 0.48 | | 0.48 | |
| Breast | 0.48 | | 0.32 | | 34.30 | | 1.89 | | 1.89 | |
| Belly | 0.42 | | 0.28 | | 45.58 | | 0.39 | | 0.38 | |
| Crown | 0.31 | | 0.31 | | 16.15 | | 0.63 | | 0.63 | |
| Nape | 0.34 | | 0.31 | | 16.72 | | 1.52 | | 1.51 | |
| Back | 0.48 | | 0.38 | | 21.16 | | 2.44 | | 2.44 | |
| Rump | 0.47 | | 0.38 | | 22.23 | | 1.54 | | 1.57 | |
| Tail | 0.34 | | 0.37 | | 16.50 | | 1.28 | | 1.28 | |
| Wing Covs | 0.38 | | 0.37 | | 16.99 | | 2.70 | | 2.65 | |
| Facial mark | 0.46 | | 0.29 | | 41.71 | | 3.31 | | 3.30 | |
| *A. strigilatus* |  | |  | |  | |  | |  | |
| Throat | 0.40 | | 0.33 | | 32.61 | | 1.75 | | 1.74 | |
| Belly | 0.36 | | 0.33 | | 37.14 | | 1.87 | | 1.85 | |
| Back | 0.30 | | 0.29 | | 18.87 | | 1.55 | | 1.53 | |
| Rump | 0.31 | | 0.31 | | 20.59 | | 2.52 | | 2.49 | |
| Tail | 0.34 | | 0.36 | | 21.18 | | 3.28 | | 3.26 | |
| Wing Covs | 0.33 | | 0.32 | | 17.29 | | 2.81 | | 2.78 | |
| *P. erythropterum* |  | |  | |  | |  | |  | |
| Throat | 0.53 | | 0.34 | | 31.23 | | 0.73 | | 0.74 | |
| Breast | 0.41 | | 0.32 | | 33.04 | | 0.48 | | 0.48 | |
| Belly | 0.42 | | 0.32 | | 32.47 | | 0.43 | | 0.42 | |
| Crown | 0.32 | | 0.29 | | 18.69 | | 0.36 | | 0.36 | |
| Nape | 0.25 | | 0.29 | | 20.49 | | 0.81 | | 0.79 | |
| Back | 0.31 | | 0.29 | | 18.13 | | 0.61 | | 0.61 | |
| Rump | 0.36 | | 0.30 | | 21.69 | | 1.23 | | 1.24 | |
| Tail | 0.36 | | 0.36 | | 18.66 | | 0.69 | | 0.67 | |
| Wing Covs | 0.39 | | 0.34 | | 18.13 | | 1.69 | | 1.68 | |
| *S. albescens* |  | |  | |  | |  | |  | |
| Throat | 0.31 | | 0.27 | | 33.07 | | 1.41 | | 1.42 | |
| Breast | 0.33 | | 0.28 | | 30.90 | | 3.99 | | 4.00 | |
| Belly | 0.39 | | 0.29 | | 37.18 | | 2.43 | | 2.45 | |
| Crown | 0.32 | | 0.32 | | 16.96 | | 1.12 | | 1.14 | |
| Nape | 0.31 | | 0.30 | | 17.55 | | 1.70 | | 1.69 | |
| Back | 0.33 | | 0.29 | | 16.78 | | 1.43 | | 1.42 | |
| Rump | 0.34 | | 0.30 | | 18.03 | | 0.59 | | 0.57 | |
| Tail | 0.30 | | 0.30 | | 14.72 | | 0.91 | | 0.92 | |
| Wing Covs | 0.36 | | 0.31 | | 16.47 | | 1.98 | | 1.88 | |
| *S. gujanensis* |  | |  | |  | |  | |  | |
| Throat | 0.34 | | 0.27 | | 38.22 | | 1.85 | | 1.86 | |
| Breast | 0.34 | | 0.29 | | 25.31 | | 1.43 | | 1.42 | |
| Belly | 0.35 | | 0.29 | | 26.06 | | 0.18 | | 0.18 | |
| Crown | 0.25 | | 0.28 | | 16.50 | | 0.70 | | 0.70 | |
| Nape | 0.28 | | 0.29 | | 16.33 | | 0.63 | | 0.62 | |
| Back | 0.30 | | 0.30 | | 16.15 | | 0.90 | | 0.90 | |
| Rump | 0.31 | | 0.30 | | 17.33 | | 1.94 | | 1.93 | |
| Tail | 0.27 | | 0.33 | | 19.03 | | 0.29 | | 0.29 | |
| Wing Covs | 0.34 | | 0.33 | | 16.69 | | 1.31 | | 1.31 | |
| *C. vulpina* |  | |  | |  | |  | |  | |
| Throat | 0.35 | | 0.26 | | 40.73 | | 2.00 | | 2.00 | |
| Breast | 0.39 | | 0.29 | | 31.18 | | 2.32 | | 2.30 | |
| Belly | 0.39 | | 0.30 | | 27.65 | | 2.73 | | 2.71 | |
| Crown | 0.36 | | 0.34 | | 18.07 | | 0.56 | | 0.55 | |
| Nape | 0.37 | | 0.33 | | 18.56 | | 1.38 | | 1.36 | |
| Back | 0.40 | | 0.35 | | 17.84 | | 1.01 | | 1.00 | |
| Rump | 0.41 | | 0.34 | | 19.77 | | 1.83 | | 1.82 | |
| Tail | 0.35 | | 0.35 | | 17.42 | | 0.93 | | 0.91 | |
| Wing Covs | 0.36 | | 0.35 | | 18.21 | | 1.64 | | 1.62 | |
